# Effect of Diet and Lifestyle Changes on Gut Microbial Diversity in Healthy Adolescents

**DOI:** 10.4014/jmb.2503.03018

**Published:** 2025-06-12

**Authors:** Juyoun Kang, Yejin Choi, Gi Beom Keum, Hyunok Doo, Jinok Kwak, Haram Kim, Yeongjae Chae, Suyoung Lee, Hyunjin Yang, Sheena Kim, Xingmin Sun, Hyeun Bum Kim, Soo Jin Yoo

**Affiliations:** 1 Department of Animal Biotechnology, Dankook University, Cheonan, Republic of Korea; 2 Department of Molecular Medicine, Morsani College of Medicine, University of South Florida, Tampa, FL, USA; 3 Department of Laboratory Medicine, Uijeongbu Eulji Medical Center, Eulji University, Uijeongbu, Republic of Korea

**Keywords:** Lifestyle changes, adolescent, gut microbiome, metagenomics, alpha diversity

## Abstract

The human gut microbiome is a complex ecosystem shaped by both intrinsic and extrinsic factors, with external elements such as diet and exercise significantly influencing its diversity and composition. In this study, we evaluated gut microbiome shifts in adolescents participating in a four-week camp with controlled diets, lifestyle, and a healthy living environment. Stool samples were collected before and after the camp period and analyzed through 16S rRNA gene sequencing to assess changes in microbial composition and diversity. Post-intervention, gut microbiome diversity increased significantly, with notable changes in the relative abundance of taxa such as *Lachnospira*, *Alistipes*, and *Barnesiella*, which are associated with enhanced immune function and gut health. Additionally, functional prediction using PICRUSt indicated an increase in genes associated with energy production and metabolism, suggesting a broader functional impact of lifestyle modifications on gut microbial functionalities. These findings revealed the potential causal relationships between lifestyle modifications and gut microbiome shifts, providing valuable insights into the interactions between environment, diet, and the gut microbiota.

## Introduction

The human gut microbiota is a complex ecological system shaped by an interaction of intrinsic and extrinsic factors [1]. The composition of the gut microbiome is highly individualized across hosts and is shaped and regulated by various factors throughout life, beginning with a unique bacterial profile established at birth. In the first 2-3 years after birth, lactation and food intake are critical for the development of gut microbial diversity, which can significantly impact the host’s health in adulthood. After a few years, the microbiota stabilize into a core gut microbial profile, maintaining the relative proportions of taxa [2].

Many extrinsic factors influence gut microbial diversity and composition, including diet, exercise, medications (such as antibiotics), and the surrounding environment, while diet is known to have the greatest impact [3]. Diet has been reported to account for more than 20% of the variation in human microbiota composition, in combination with anthropometric measurements [4]. This suggests that modulating gut microbiota through dietary changes holds potential for health promotion and disease management. A study in which African Americans and rural Africans switched diets for two weeks revealed significant differences in gut microbial communities, colonic metabolites, and mucosal proliferative markers [5]. This suggests that changes in gut microbiota driven by diet may alter intestinal mucosal status as well as overall health and immune function. Short-term, dramatic dietary interventions have been shown to rapidly alter the diversity of the human microbiome, however these changes are transient and typically last no longer than a few days [6]. Even after significant dietary changes, an individual's microbiome retains its unique and personalized composition, suggesting that the factors controlling ecological homeostasis extend beyond dietary influence [7].

Lifestyle factors such as diet, exercise, and environment represent an important and generally accessible natural means of controlling the nutrients supply to the intestinal microorganisms [1]. Although many studies showed that dietary interventions can specifically modulate gut microbiome composition, further progress in this approach is complicated by interindividual variability of the microbial community response [8]. The reported causes of this variability include the baseline microbiome composition features, but it is unclear whether any of them are intervention specific. Studying the effects of diet on gut microbiota diversity is complicated by interindividual variability (genetics and geography), host factors (age, medication use, and health condition), temporal fluctuations, the complexity of diet, and compliance, making it hard to isolate dietary influences from other factors [3, 9]. Many studies have tested specific diets to observe changes in microbial composition and diversity. However, control of variables such as baseline diets, lifestyle, and environmental factors was limited in these studies [10, 11].

Therefore, we evaluated gut microbiome shifts in adolescents participating in a four-week camp with controlled diets, lifestyle, and a healthy living environment. With informed consent, we collected stool samples before and after the camp to assess the extent to which changes in environment and diet influenced alterations in the gut microbiome. Our study provided a unique opportunity to observe the impact of lifestyle, especially dietary changes, on the intestinal microbiome within a group sharing the same environment, daily activities, and diet during nearly a month. While genetic factors, health status, and baseline individual intestinal microbial compositions could not be entirely excluded, we minimized external factors and provided a controlled diet, allowing us to assess the impact of short-term changes on the gut microbiome.

## Materials and Methods

### Subjects

Volunteers were recruited from healthy Korean adolescents who participated in a summer camp. All participants were asked about their age, gender, area of residence, height, weight, underlying disease, allergies, medication intake, dietary supplements including probiotics, eating habits, exercise, and sleeping habits. Exclusion criteria included acute illnesses, chronic underlying diseases, recent antibiotic use, or significant weight change within the three months prior to recruitment.

This camp took place at a forest campground for four weeks during the summer of 2023. Over the four-week period, 84 meals (28 each for breakfast, lunch, and dinner) and 28 nightly snacks were provided, along with daily outdoor physical activities. The camp's meals were prepared in-house using fresh organic ingredients, with the intention of offering a balanced diet that included plenty of dietary fiber and fermented foods. Nutrient sources and calorie supply were analyzed based on the average intake of each meal.

### Ethical Considerations

Participation in this study was not affiliated with the camp. Adolescents were included in this study only when both they and their parents agreed to participate with informed consent provided by the parents. Compared to other camp participants, those in the study had no additional interventions beyond submitting stool samples. We obtained ethical approval from the institutional review board of Sanggye Paik Hospital (Approval No. 2023-10-011).

### Sample Collection

Stool samples were collected within 3 days before the start of camp (Pre group) and within 3 days after its end (Post group). After being sealed in a sterile container, it was sent to the laboratory within 6 hours, kept on ice pack, and stored in a -70°C deep freezer until analysis.

### 16S Metagenomic Analysis

The gut microbiome was analyzed using 16S rRNA gene sequencing of stool samples collected from participants. Total DNA of the fecal samples was extracted using a QIAamp PowerFecal Pro DNA Kit (Cat. #51804; Qiagen, USA), according to manufacturer’s instructions. Diluted total fecal DNA (5 ng/ml) was used for PCR amplification with a universal primer set targeting from the V3 to V4 region of the 16S rRNA gene (341F, 5’-CCT ACG GGN GGC WGC AG-3’; 805R, 5’-GAC TAC HVG GGT ATC TAA TCC-3’). The amplicon library preparation was performed using IDT 16S V3-V4 package kit (Integrated DNA Technologies, USA) and the amplicons were sequenced using the Illumina MiSeq platform (2×300 bp paired-end reading method) (Sanigen, Inc., Republic of Korea).

### Bioinformatic Analysis

Paired-end sequencing reads were merged using the MicrobiomeHelper software package, and analysis was conducted through the QIIME2 pipeline. Sequence quality was enhanced by filtering based on Phred quality scores and denoising with the deblur algorithm. The generated amplicon sequence variants (ASVs) were taxonomically assigned using a naïve Bayesian classifier trained on the RDP reference database. Alpha diversity was calculated using the Observed features, Chao1, Shannon, and Simpson indices, and statistical differences between the pre-samples and post-samples were assessed using the Mann-Whitney test. Beta diversity was calculated using Weighted and Unweighted UniFrac Distance Metrics, and differences in microbial community composition between the groups were evaluated using ANOSIM. Significant differences in the relative abundance of specific taxa between the Pre group and Post group were identified using White’s non-parametric t-test with STAMP software. Functional annotation of the predicted metagenomes was performed using the Kyoto Encyclopedia of Genes and Genomes (KEGG) pathways. Genes were classified into various KEGG Orthology (KO) levels to enable a detailed exploration of microbial functions, and significant differences were evaluated using Welch's t-test.

### Data Availability

The data set of this study has been deposited in the NCBI repository under the accession number PRJNA1210156.

## Results

### Subjects

A total of 13 adolescents participated in this study. They were 10 to 15 years old (average 13.1 years), and 8 were male (61.5%). Regarding daily lifestyle habits, they answered that they regularly exercise (9 out of 13, 69.2%), eat fermented foods (12 out of 13, 92.3%), and drink fermented beverages like yogurt (3 out of 13, 23.1%). A total of 23 samples were collected, including 10 paired samples submitted before and after the camp, one sample collected before the camp, and two samples collected after the camp. After the camp, their height and weight increased slightly, however the change was not significant (Table 1). During the camp, they exercised more frequently and for longer durations than in their previous lifestyle and consumed more fermented foods compared to their daily diet before the camp.

### Analysis of Diet during Camp

All meals were prepared on-site using organic ingredients. Breakfast typically consisted of sourdough bread made from fermented dough with freshly ground whole grains of organic ancient wheat, fermented kefir milk, an egg dish (scrambled, fried, boiled, or sous-vide), and one or two fruits, such as apples, grapes, blueberries, bananas, or peaches. On four occasions, rice, scones, or waffles were served in place of sourdough bread, and on those days, sourdough bread was served at lunch instead. Grains (rice, bread, or noodles) and vegetables were included in every lunch and dinner. Nearly all lunches and dinners (except four lunches) featured at least one protein source: meat (beef, pork, chicken), fish, tofu, or egg. Fruits were served 20 out of 28 lunches and dinners. Nightly snacks included baked goods such as bread, cookies, and rice cakes on 15 occasions, dairy products like milk and yogurt on 15 occasions, and fruit on 10 occasions. Green tea was served almost every day. Daily nutritional support, based on recommended amounts for their age, averaged 2,467 kcal/day (2500 for boys, 2000 for girls), with carbohydrate intake at 296.8 g/day (recommended: 130 g/d), protein at 107.4 g/day (recommended: 60 for boys, 55 g/day for girls), and fat at 94.6 g/day. The calorie sources were divided among carbohydrate, protein, and fat at 34.5%, 17.4%, and 48.1%, respectively, compared to the recommended ranges of 55-65%, 7-20%, and 15-30%, respectively.

### Microbiome Analysis

The diversity and composition of the gut microbiota were analyzed among camp participants who maintained a consistent lifestyle and diet. After merging the sequences, each sample yielded between 52,990 and 381,225 reads, with a total of 5,133,407 reads identified. Following the Deblur process, 4,072 features were generated.

**Fig. 1:**
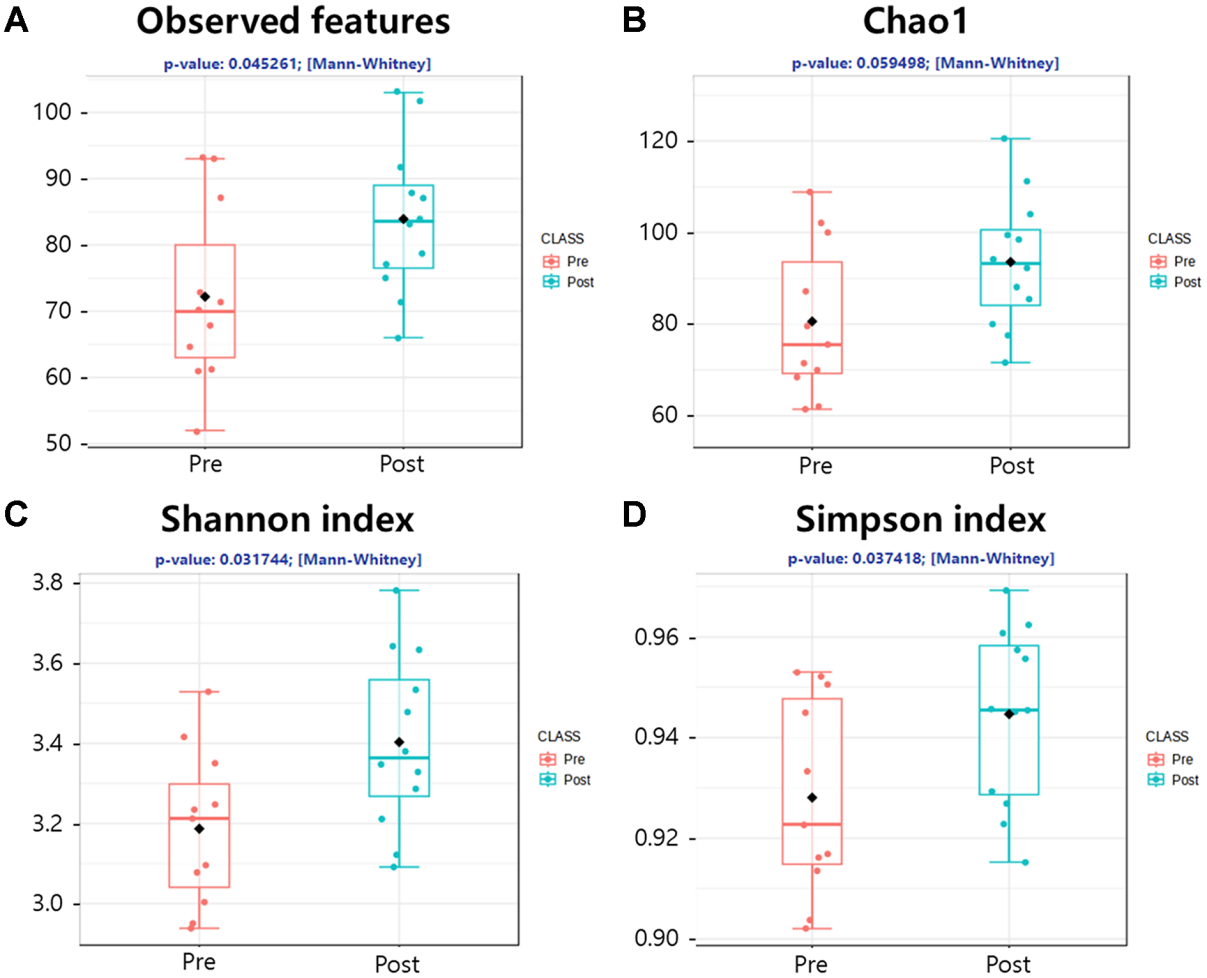
Box plots showing the alpha diversity indices of the microbiome in adolescent fecal samples collected before (Pre) and after (Post) the camp. (**A**) the number of observed operational taxonomic units (OTUs), (**B**) the chao1 diversity index (*P* = 0.059), (**C**) the shannon diversity index (*P* = 0.032), and (**D**) the simpson diversity index (*P* = 0.037). In each plot, the box represents the interquartile range (IQR) spanning the 25th to 75th percentiles, while the horizontal line within the box indicates the median value.

**Alpha diversity.** Operational Taxonomic Units (OTUs) were clustered at 97% sequence identity for each sample to compare microbial diversity and species richness between the Pre and Post groups. Alpha diversity was assessed using indices that estimate species richness (Observed features and Chao1) and evenness (Shannon and Simpson indices) (Fig. 1). These microbial diversity indices showed significantly higher species diversity in the Post group for the Observed features, Shannon, and Simpson indices (*P* < 0.05) compared to the Pre group with borderline significance for the Chao 1 index (*P* = 0.059).

**Fig. 2:**
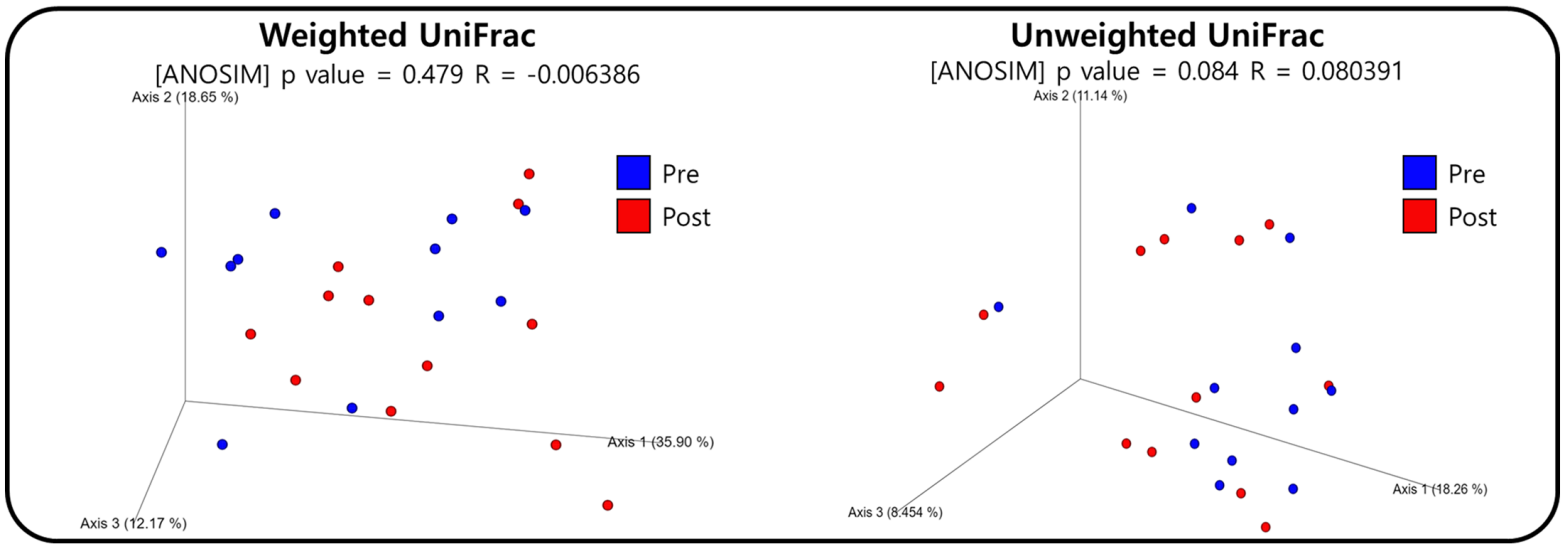
Principal coordinate analysis (PCoA) plots based on weighted and unweighted UniFrac distance metrics. Pre camp samples are shown in blue, and post-camp samples are shown in red, each represented as individual points.

**Beta diversity.** Beta diversity was visualized using PCoA plots based on Weighted and Unweighted UniFrac distances to depict the microbial community structure in quantitative (weighted) and qualitative (unweighted) terms (Fig. 2). The analysis of microbial community similarity using ANOSIM showed an R value below 0.1, indicating no significant difference between the two groups.

**Fig. 3:**
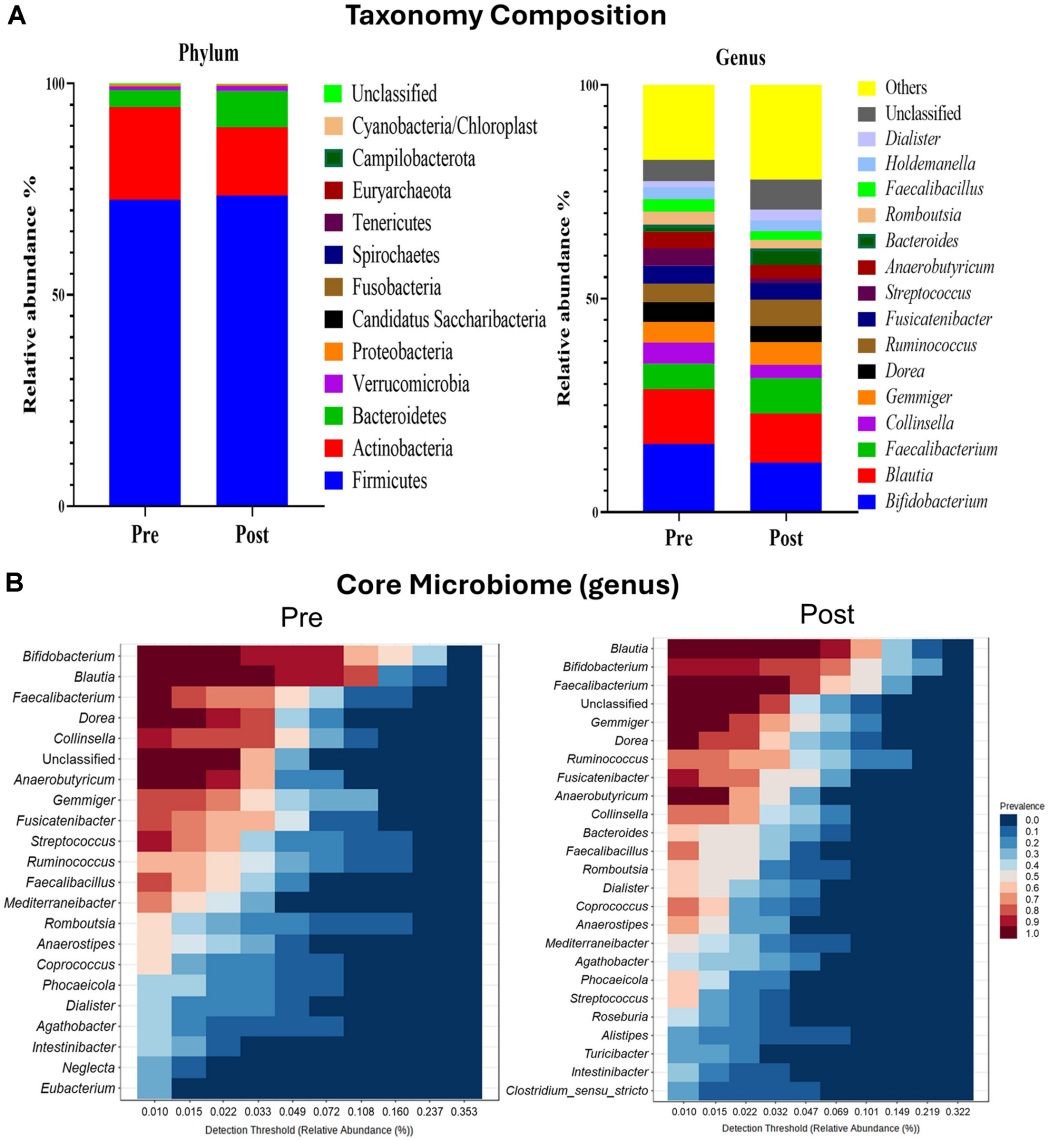
(A) Taxonomic classification of microbiota shown as stacked bar plots at phylum and genus levels, (B) a heatmap depicting the core microbiome at the genus level.

**Fig. 4:**
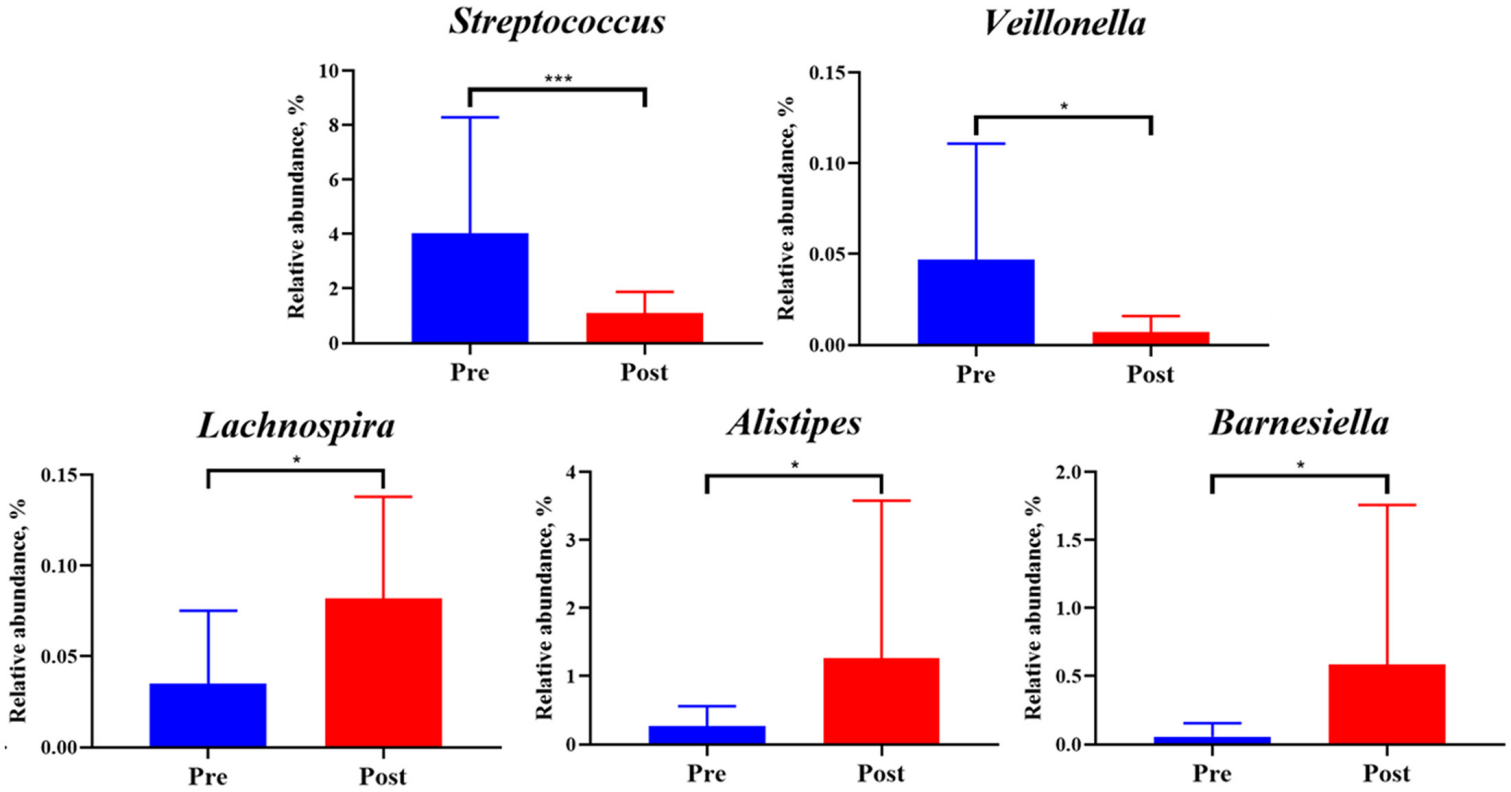
The relative abundances of five genera between Pre and Post groups consuming the diet designated by the camp, as analyzed using STAMP (***p < 0.001, *p < 0.05).

**Taxonomic classification.** Taxonomic classification based on the RDP database illustrated the fecal microbial composition at different taxonomic levels for the Pre and Post groups (Fig. 3A). At the phylum level, both groups were composed of Firmicutes, Actinobacteria, and Bacteroidetes. Firmicutes, which accounted for the largest proportion, ranged from 55.84% to 97.15% in the Pre group, with an average of 72.49%, and increased to 73.48% in the Post group. Actinobacteria decreased from 21.91% in the Pre group to 16.09% in the Post group, whereas Bacteroidetes saw an increase from 4.02% to 8.68% following the intervention. At the genus level, the predominant genera were *Bifidobacterium*, *Blautia*, and *Faecalibacterium*, which were consistently represented in both Pre and Post groups. *Bifidobacterium* and *Blautia* accounted for over 10% of the microbial community in both periods, and their relative abundance decreased in the Post group compared to the Pre group. Conversely, *Faecalibacterium* exhibited an increase in relative abundance of the Post group. Overall, while there were minor shifts in the relative abundances of specific taxa, the dominant taxa consistently maintained a high proportion of the microbiome. The analysis of the core microbiome revealed that, when considering both relative abundance and prevalence, the dominant taxa remained consistently prominent across the majority of individuals (Fig. 3B). A two-sided Welch’s *t*-test in STAMP was used to confirm significant differences in the relative abundances of taxa between Pre and Post groups, and these differences were visualized using an extended error bar plot. The comparison of the camp lifestyle Pre and Post groups revealed that the relative abundances of *Streptococcus* and *Veillonella* decreased, whereas *Lachnospira*, *Alistipes*, and *Barnesiella* exhibited a statistically significant increase in the Post groups (Fig. 4).

**Fig. 5:**
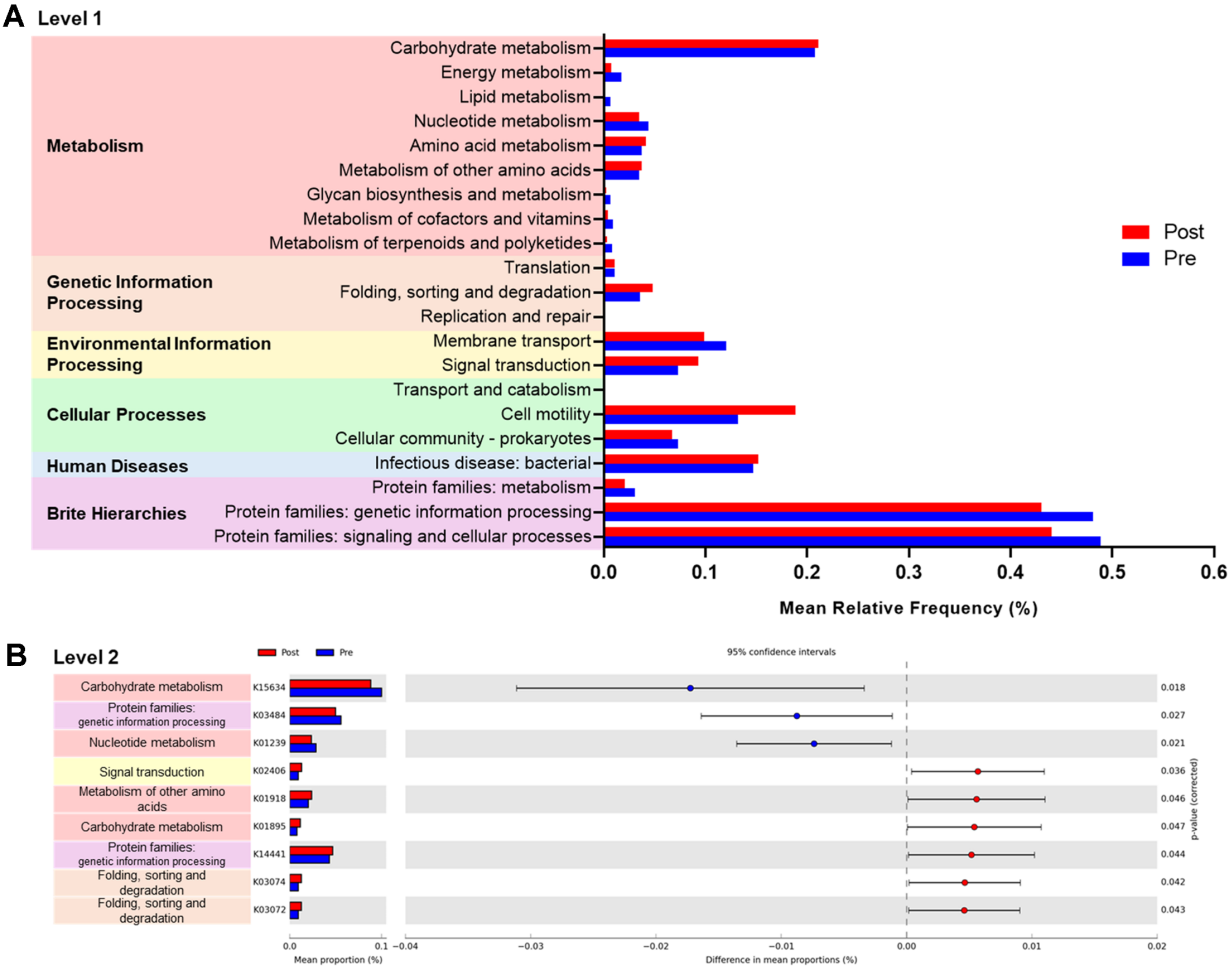
PICRUSt analysis results of predicted functional pathways in the gut microbiota. (**A**) Relative abundances of Level 1 and 2 functional categories with significant differences between Pre and Post groups. (**B**) Extended error bar plot showing significant differences in specific gene-level predictions, analyzed using the STAMP software.

**Predictive metagenome analysis.** The functional predictions of gut microbiota under environmental influences were inferred using PICRUSt and KEGG database pathways. Among the 6208 KO genes mapped to 266 KEGG pathways, 321 KO genes were found to differ significantly, as analyzed using Welch’s *t*-test in the STAMP software. At the Level 1 category, a notable observation was the increased relative abundances of “Genetic Information Processing” and “Cellular Processes” in the Post group compared to the Pre group (Fig. 5A). Focusing on key KO genes, K01895 in the “Carbohydrate Metabolism” category, K01918 in the “Metabolism of Other Amino Acids” category, and K14441 in the “Genetic Information Processing” category exhibited significantly higher predicted relative abundances in the Post group (Fig. 5B).

## Discussion

Several studies have examined changes in gut microbiota in human populations in response to dietary or lifestyle changes, but it is difficult to control all consumed items, including snacks and drinks [12-14]. Additionally, fully controlling external factors like living environment, exercise, and outdoor activities is challenging [15, 16]. However, this study observed gut microbiota changes while minimizing variables by analyzing microbial composition and diversity before and after communal living, where participants shared the same environment, spaces, activities, and identical meals and snacks. Furthermore, since all participants were healthy teenagers, limitations related to illness or health problems were excluded. Although the study was conducted over a short period, it confirms that lifestyle changes such as dietary and environmental modification can positively affect gut microbiota diversity, along with beneficial changes in the relative abundance of several genera.

This observational study involved teenagers participating in a four-week healthy lifestyle camp, with only those who consented providing fecal samples. The camp involved a shift from urban South Korea to rural Canada, where students who previously spent over six hours daily in school engaged in daily outdoor activities. Dietary changes included an increase in fermented foods and dietary fiber compared to the pre-camp diet. Fermented foods were provided daily through whole-grain sourdough bread and kefir fermented milk, while dietary fiber came from sourdough bread, salads, fresh vegetables, and fruits. Additionally, green tea was consumed daily.

A key finding from this study was the significant increase in alpha diversity indices, including species richness and evenness, in the Post group. Greater gut microbiome diversity has been associated with enhanced metabolic functions, improved immune response, and reduced susceptibility to diseases such as obesity and inflammatory bowel disease [1]. The significant increase in alpha diversity can be attributed to the participants' exposure to a high-fiber, fermented, and organic diet [17, 18]. In contrast, beta diversity analysis using PCoA plots revealed no significant difference between the Pre and Post groups, with an ANOSIM R-value below 0.1. This indicates that while alpha diversity increased, the overall structure of the microbial communities remained stable. Such stability underscores the resilience of the gut microbiome to short-term dietary and environmental modifications, consistent with previous findings [3, 7]. The core microbiota also remained stable despite changes in microbial diversity, suggesting that host-specific microbial communities are resistant to transient environmental changes. This highlights the importance of long-term dietary adherence for sustained impacts on gut microbiome composition, as short-term interventions may lead to only temporary changes.

Taxonomic analysis showed a reduction in Actinobacteria and an increase in Bacteroidetes at the phylum level. At the genus level, the relative abundances of *Streptococcus* and *Veillonella* decreased, while *Lachnospira*, *Alistipes*, and *Barnesiella* increased. The decline in *Streptococcus* and *Veillonella*, which are typically found in the human oral cavity and gut, may reflect improved healthy microbiome, as these genera are associated with pathogenicity under certain conditions [19]. *Streptococcus* includes species linked to respiratory diseases, sepsis, and inflammation, while *Veillonella* is a general commensal but has been implicated in polymicrobial infections [20, 21]. Conversely, the increased abundance of *Lachnospira*, known for producing short-chain fatty acids (SCFAs) such as acetate, propionate, and butyrate, supports its anti-inflammatory and health-promoting properties [22]. Previous studies have also linked *Lachnospira* to high dietary fiber intake, suggesting a positive association with dietary intervention. Similarly, *Barnesiella* plays a beneficial role in carbohydrate fermentation, immune modulation, and the inhibition of pathogenic bacteria [23]. *Alistipes*, predominantly found in healthy gut microbiota, has been reported to protect against colitis and liver cirrhosis, further underscoring the potential health benefits of these taxonomic shifts [24, 25].

The functional analysis of the gut microbiome showed a significant increase in K01895 (acetyl-CoA synthetase) within the “Carbohydrate metabolism” category after the camp. This indicates enhanced SCFA production, particularly acetate, which is known to support intestinal barrier integrity and modulate immune responses [26]. In addition, the increased abundance of K01918 (Pantoate-beta-alanine ligase) in the “Metabolism of other amino acids” category suggests a potential enhancement in vitamin B5 biosynthesis, an essential component for fatty acid metabolism [27]. The elevation of K14441 (ribosomal protein S12 methylthiotransferase) in the “Genetic Information Processing” category may contribute to microbial health by promoting the growth and stability of beneficial gut bacteria [28].

Dietary interventions, including high-fermented foods and high-fiber diets, have demonstrated significant effects on the gut microbiota and its associated metabolic functions. Fermented foods, such as fermented tea, sauerkraut, soy milk and kimchi are known to enhance the abundance of beneficial bacterial taxa, including *Lactobacillus* and *Bifidobacterium* depending on the specific food type and fermentation process [29]. Sourdough bread, a high-fiber fermented food, has been shown to promote *Bifidobacterium* growth [30]. High-fiber diets, consisting of whole grains, vegetables, fruits, and legumes, provide complex carbohydrates that are indigestible in the upper gastrointestinal tract but serve as substrates for microbial fermentation in the colon. This fermentation process produces SCFAs and supports the proliferation of beneficial genera, such as *Faecalibacterium*, *Ruminococcus*, *Akkermansia*, and *Roseburia* [31, 32]. Increased intake of fresh vegetables and fruits also influences colonic microbiota, as these foods carry distinct microbiomes that reflect their plant species and can transfer microorganisms to the human gut [33, 34]. Once introduced to a new host, these microbes adapt based on factors like pH, temperature, and competition with resident microbes, allowing some to persist while others remain transient [35].

The impact of physical activity on gut microbial diversity and composition has been observed in comparative studies between obese and non-obese children. In obese individuals, the gut microbiota shows a reduction in Bacteroidetes, *Bifidobacterium*, and alpha diversity, while being enriched in Proteobacteria and *Lactobacillus* [36]. The effects of physical activity have been known to improve alpha diversity and promote the growth of beneficial bacteria linked to weight loss in children and adolescents. These beneficial changes include a decrease in Proteobacteria and an increase in Firmicutes, such as *Blautia*, *Dialister*, and *Roseburia* [37].

This study effectively controlled external variables by having adolescents share the same living environment, diet, and physical activity, allowing for the observation of gut microbiota changes under consistent conditions. As a result, the high-fiber, fermentation-rich diet and regular physical activity were shown to increase gut microbiome diversity, significantly enhance the proportion of beneficial taxa, and improve metabolic potential in functional analyses, demonstrating the impact of lifestyle modifications on gut health.

## Figures and Tables

**Table 1:** Diet and daily lives baselines and study periods.

	Baseline	During or after camp	P value
BMI (kg/m^2^)	18.8 (4.3)	18.7 (3.9)	0.750
Height (cm)	162.1 (9.5)	163.1 (9.7)	0.793
Weight (Kg)	50.6 (16.5)	50.7 (15.5)	0.964
Exercise (times/day)	0.4 (0.3)	1.8 (0.7)^a^	<0.0001
Exercise (hours/day)	0.3 (0.4)	1.8 (1.2) ^a^	0.0003
Fermented food (g/day)	197.8 (101.7)	537.0 (337.6) ^a^	0.0020
Fermented drink (mL/day)	17.0 (34.9)	179.6 (72.4) ^a^	<0.0001
Bristol stool scale (median, IQR)^b^	3 (2.25, 4)	3.5 (2.75, 4)	0.305

Continuous variables reported as mean ± S.D. or median (IQR). Categorical variables reported as N (%). ^a^These mean values indicate the average frequency of daily exercise and the daily amount of food served during the camp. ^b^The Bristol Stool Scale evaluates stool type based on shape and consistency, with scores of 3-4 indicating neither constipation nor loose stools.
